# Notes on Dermatology

**Published:** 1894-10

**Authors:** Isadore Dyer

**Affiliations:** New Orleans, La., Professor of Dermatology in the New Orleans Polyclinic; Lecturer and Clinical Instructor in Skin Diseases, Medical Department Tulane University, Etc.


### NOTES ON DERMATOLOGY.

BY ISADORE DYER, M. D., NEW ORLEANS, LA.,
Professor of Dermatology in the New Orleans Polyclinic; Lecturer and Clinical Instructor in Skin Diseases, Medical Department Tulane University, etc.

MASSAGE IN THE TREATMENT OF PRURIGO.—R. Hatsche, of Vienna, reports eleven cases of prurigo benefited by this procedure. The itching was relieved, and the cases cured, in two or three weeks. The rubbing was done daily, for fifteen or twenty minutes at first, and gradually reduced as the symptoms diminished. The method employed was the simple rubbing, starting from the distal end of the extremities and working toward the center.—*Journal of Cutaneous and Gen.-Urin. Dis.*, for June, 1894.

---

TRICHOMYCOSIS CIRCINATA is the classical term now applied to the ringworm group. Dr. Sabouraud distinguishes three different forms of this affection. One is the trichomycosis, with minute sporules, caused by the microsporon audouini. The diseased hairs are fine, greyish, all bent in the same direction, and broken off six or seven millimeters from the scalp. A large number of ashen scales are scattered over the diseased area, constituting what is known as pityriasis alba parasitica. The microscope shows the hair sheath made up of small, closely packed spores, placed side by side. A second variety is due to the trichophyton tonsurans, of human origin. Here the hair is large, dark colored, short, and bare. It is not sheathed, and there is no epidermic lesion about it. Under the microscope, the spores, which are comparatively large, are contained *within* the hair, and are arranged in regular chains along its longitudinal axis.

The trichophyton of animal origin, which comprises about one-twentieth of all cases of ringworm, may be due to various species of parasites, all having the common characteristic scaly lesions of epidermis. The spores vary in size, and are arranged in longitudinal rows. This last variety is of shorter duration than the other two, seldom exceeding three or four months. The small spore variety may last two years or more.

The treatment depends upon the variety. In all cases, the treatment is instituted with the tincture of iodine. This stains the parasitic rings, and outlines the diseased areas. Epilation, washing the scalp, and thorough attention to cleanliness, still is recommended. In the barber's itch variety, the treatment at the start may have to be mild, owing to the inflammation. Here starch poultices are suggested at the beginning, until the acute condition is relieved. Then iodine ointments are applied, beginning with three per cent, and increasing until the pure tincture can be applied. This is supplemented with mercurial plaster. In those cases of trichomycosis circinata with small spores, Dr. Sabouraud uses, instead of this mercurial plaster, the following:

℞ Potassii carbonatis................ʒ ijss

Aquæ destillat.

Ol. amygdalæ dulcis ........aa gr. lxxv

Ung. Petrolati ......................ʒ x

M. Sig.: Apply for twenty-four hours.

After this has been applied for the time specified, the scalp is soaped and washed, and then painted with tincture of iodine. In this manner, the treatment is continued.

